# Human Corneal Endothelial Cell Cultivation From Old Donor Corneas With Forced Attachment

**DOI:** 10.1038/s41598-017-00209-5

**Published:** 2017-03-10

**Authors:** Mohit Parekh, Sajjad Ahmad, Alessandro Ruzza, Stefano Ferrari

**Affiliations:** 1 International Center for Ocular Physiopathology, The Veneto Eye Bank Foundation, Venice, Italy; 2 Department of Molecular Medicine, School of Biomedicine, University of Padova, Padova, Italy; 3 Moorfields eye hospital, London, UK; 4 Institute of Ophthalmology, University College London, London, UK

## Abstract

Human corneal endothelial cells (HCEnCs) are responsible for maintaining the transparency of the cornea. Damaged or diseased HCEnCs may cause blindness. Replacement of the diseased cells with a healthy donor endothelium is the only currently available treatment. Tissue-engineering can serve as an alternative to conventional donor corneal transplantation. Due to the global shortage of donor corneas, a wide interest in the development of cultured graft substitutes and artificial corneas has increased. Availability of the old donor corneas is higher especially for research. Although it can be proposed as a valuable source for cell culture, its less proliferative capability emerges a challenge for the researchers. This article describes the use of hyaluronic acid (HA) in combination with Rho-kinase inhibitor (ROCK) Y-27632 for the cultivation of HCEnCs from older donor corneas (age > 60 years). Four conditions including and excluding HA + ROCK and its effect on early attachment rates and proliferation was studied on forty-eight corneas. It was observed that HCEnCs reach confluence within 10–15 days when cultured with HA + ROCK. This approach improves the efficiency of cell adhesion due to force attachment. HCEnCs from old donor corneas can be cultured using this method which may further lead to cell-based therapy for treating corneal endothelial dysfunction.

## Introduction

Human cornea is made of several layers. The posterior endothelial monolayer is responsible for maintaining the required transparency of the cornea. An osmotic gradient is generated by the transmission of essential metabolites across the corneal endothelium, which transports water into the cornea. The corneal endothelium continuously pumps the water, ions and solutes out of the cornea using trans-membrane ion channels^1^. Increased water content in the cornea can lead to oedema and hence opacity which is responsible for corneal blindness^2^. Human corneal endothelial cells (HCEnCs) maintain the clarity and thickness of the cornea^3^. Endothelial failure is seen mostly as a cause of Fuch’s endothelial dystrophy, which is one of the common reasons for corneal endothelial replacement.

Penetrating keratoplasty (PK) is the most popular choice among the surgeons to treat endothelial disorders. However, with the recent advancements, endothelial keratoplasty (EK) has shown clinically relevant results like early rehabilitation rate and better visual outcome over PK and is gradually been accepted by the surgeons due to standardized procedures^4^. The only recognized treatment for endothelial disorders so far is a corneal replacement. However, due to the donor shortage, the transplantation options also remain limited. Therefore, alternative therapeutic approaches are currently explored to provide a worldwide solution.

One of the most common approaches for therapeutic treatment and HCEnCs regeneration includes the use of Rho-Kinase (ROCK) inhibitor for the development of allogeneic *ex vivo* expanded HCEnCs for transplantation^5^. It has been previously reported that ROCK inhibitor (Y-27632) allows adhesion of HCEnCs to a substrate and the inhibition of ROCK signalling may manipulate cell adhesion properties^6–8^. As the host endothelium is already abnormal in Fuch’s dystrophy, a direct injection of ROCK inhibitor may not be considered as a therapeutic approach, as it needs a complete replacement. However, *ex*-*vivo* expansion using ROCK inhibitor may allow potential cell-based therapy. It has been reported that despite the limited regenerative potential *in vivo*, HCEnCs have a capacity to proliferate *in vitro* ^1^. Therefore, cultured HCEnCs could be a prospective alternative treatment for corneal endothelial diseases. Several methods to define the media, conditions, isolation techniques etc for cultivation of HCEnCs have been successfully investigated so far^6, 9^.

Hyaluronic Acid (HA) binds to and protects the corneal endothelial cells. HA dissolves in saline and can be aspirated easily out of the endothelium. A thin layer of HA, however, remains on the endothelial cells^10^. The hypothesis of the present study is that the viscosity of HA is higher than the media used to culture the HCEnCs. Therefore, using gravitational pull and viscosity of HA^11^, the isolated primary HCEnCs can be pushed towards the base and allowed to be strongly adhered for culturing HCEnCs when topically applied over the cells.

Previously reported studies on culturing HCEnCs have been performed on younger donor corneas^12^. It has been observed that young donors have a high proliferative capability compared to older donor corneas. However, it is difficult to obtain young donors for culturing HCEnCs *in vitro* due to its characteristics that are suitable for transplantation. Most of the old donor corneas are easy to obtain for research due to its endothelial cell density that is less than the threshold required for transplantation. The proliferative capability is also noticed to be less. It is a challenge to culture old donor corneas for various reasons. However, if the HCEnCs from the older donors can be cultured then the availability of the source will be much higher compared to the younger donor corneas. The paper thus highlights four different conditions to identify the role of HA and Rho kinase (ROCK inhibitor) for force adherence in culture of HCEnCs which may eventually lead to higher number of corneal endothelial sheets from older donor corneas, reducing the requirement of human corneal tissues globally.

## Results

### Donor characteristics and plating density [n = 48, twenty four pairs]

Recorded average age of the donors was 63.94 (±13.79; Min-48, Max-79) years comprising of 14 Males and 10 Females. The average post mortem time was 16.71 (±6.37; Min – 5.0 h, Max – 25.35) hours. The tissues were preserved in the tissue culture medium for 31.69 (±6.67; Min – 20, Max – 40) days. Average endothelial cell density before isolation was 1943.75 (±222.02; Min – 1800, Max – 2100) cells/mm^2^ without any trypan blue positive cells (TBPCs). 92,313.58 (±10,544.16; Min – 75,988, Max – 99,734.5) cells in average per well was plated after isolation in Labt-Tek II chamber slides (8 chambers, 25 × 75 mm, 0.7 cm^2^ culture area) from Thermo Fisher Scientific (Rochester, NY, USA).

### Adhesion of HCEnCs using Hyaluronic Acid (HA)

**Figure 1:**
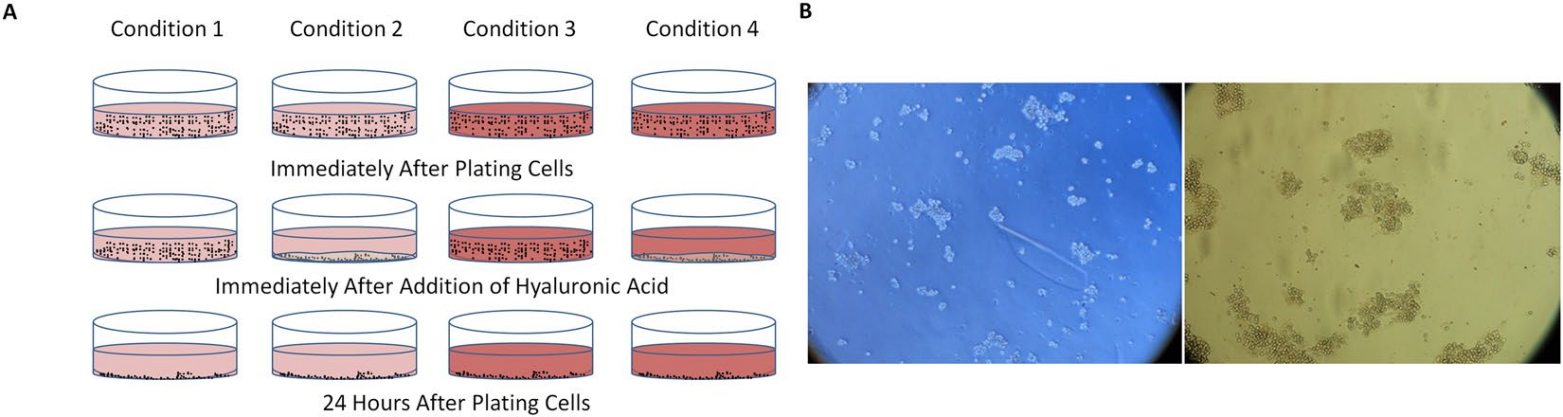
Representation of the attachment pattern at different time points and conditions. (**A**) Schematic representation of the culture conditions and attachment using hyaluronic acid (HA). It was observed that the cells attach to the plate very quickly and firmly when HA is topically applied over the cells. (**B**) left image - the cells when they are free floating in the media which are not prominently round and are at different focal plane however, right image - the cells when treated with HA shows clear borders and round shape which are observed at 100X magnification.

A schematic representation of the forced adherence using HA is shown in Fig. 1A. It is observed that the free floating cells are forcefully pushed towards the base of the FNC coated plate when HA is applied topically. It is difficult to observe the cell shape and size precisely in one plane when they are free floating compared with when they are attached on the flat surface which is observed in Fig. 1B. The free floating cells can be observed but without clear shape and borders of the cell however, when the cells are forcely adhered, they show clear cell borders and round shape.

### Culture of HCEnCs and their confluency patterns show higher proliferation from C4 [n = 30, fifteen pairs]

**Figure 2:**
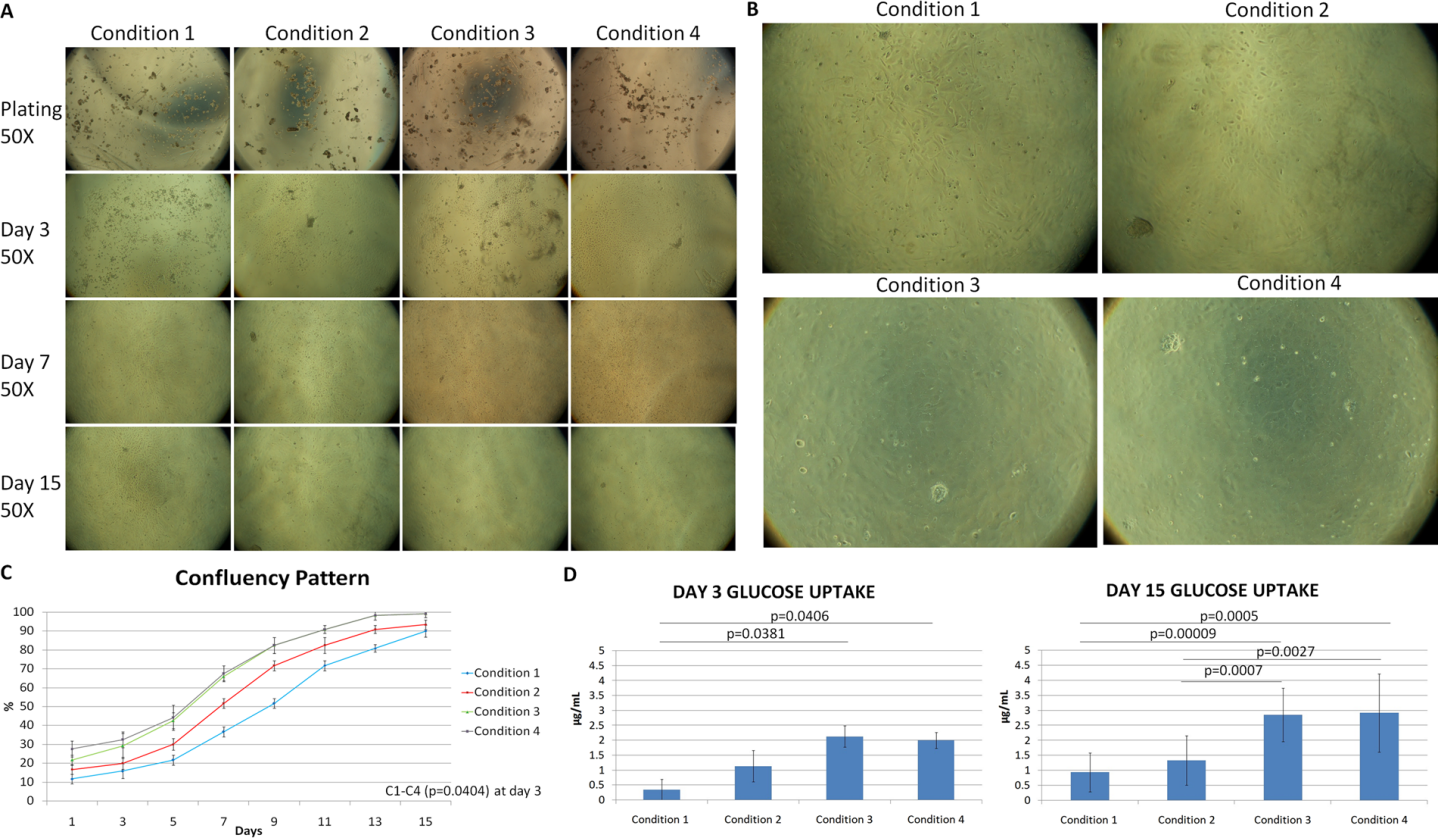
Morphological and glucose analysis. (**A**) It was observed that the cells attach firmly in C2, C3 and C4 and start proliferation within day 3. However, by day 7, C3 and C4 show a higher number of cells and growth rate. By day 15, confluence was observed in C3 and C4 and >90% of the culture was confluent in C1 and C2 at 50X magnification. (**B**) At day 15, the cell shape and size along with confluence is better and higher in C3 (bottom left) and C4 (bottom right) as seen at 100X magnification compared to C1 (top left) and C2 (top right). (**C**) The growth pattern determined that C3 and C4 show higher proliferation capability when firmly adhered to the base. Confluence of the cultured HCEnCs showed up to 30% boost in C3 and C4 compared to around 20% in C1 and C2 in early days. However, by day 15, C3 and C4 reached 99% confluence whereas C1 and C2 were at 90%. Statistical significant difference was only observed between C1–C4 (p = 0.0404) at day 3. (**D**) Higher glucose uptake was observed in C3 and C4 at day 3 and also at day 15 due to high number of metabolically active cells. Statistical significance was observed between C1–C4 (p = 0.0406) and C1–C3 (p = 0.0381) at day 3, however, at day 15 significant difference was observed between C2–C3 (p = 0.0007), C1–C4 (p = 0.0005), C1–C3 (0.00009) and C2–C4 (p = 0.0027) showing high metabolism in groups especially C3 and C4.

HCEnCs were observed at different days of culture (Fig. 2A). The cells were not passaged as only the adhesion and proliferation capability was studied and the cells were sacrificed for the immunostaining analysis at day 15. At day 3, the growth rate (%) observed in C1 (condition 1 – no HA or ROCK), C2 (condition 2 – only HA), C3 (condition 3 – only ROCK) and C4 (condition 4 – HA + ROCK) was 15.8, 20, 29.2 and 32.5, however the growth rate (%) at day 7 increased to 36.6, 51.6, 65.8 and 67.5 respectively without any statistical significance (p < 0.05). The data reveals that when the cells are adhered forcefully, they get a boost start. The cell attachment to the matrix or its base is highly important for culture and proliferation. At day 15 the cells showed (%) 90, 93.3, 99.16 and 99.16 confluency. The morphology of the cells at 100X magnification can be observed in Fig. 2B. It was observed that at day 3, C1–C4 showed statistical significance in growth rate (p = 0.0404) and the other conditions did not show any significance (Table 1). Although statistically insignificant (p > 0.05) at day 15 (Table 1), the cells showed faster proliferation in C4 in the first week of culture. Once the cells get adapted to the environment, they grow normally. It was observed that the proliferation capability of the cells after 7 days in C3 and C4 was the same. C1 and C2 took a longer time to adapt and reached almost 90% confluence at day 15 (Fig. 2C). At day 15, the cells were counted using an eye-piece reticule and in average 2330 (±115.9), 2340 (±69.9), 2480 (±131.6) and 2500 (±94.2) cells/mm^2^ were counted from C1, C2, C3 and C4 without any statistically significant difference.

**Table 1:** Statistical data comparing different conditions and parameters.

pValues	C1–C2	C2–C3	C3–C4	C1–C4	C1–C3	C2–C4
**Confluence day 3**	0.2339	0.2789	0.4294	*0.0404*	0.1459	0.0751
**Confluence day 15**	0.5495	0.5712	0.9198	0.1985	0.4922	0.5775
**Glucose uptake day 3**	0.2379	0.1763	0.7251	*0.0406*	*0.0381*	0.2185
**Glucose uptake day 15**	0.197	*0.0007*	0.888	*0.0005*	*0.00009*	*0.0027*
**% Hexagonality day 15**	0.5077	0.3357	0.2078	*0.0488*	0.2122	0.0758
**% Polymorphism day 15**	0.3209	0.2145	0.1239	*0.0268*	*0.0419*	*0.0141*
**Ki-67 day 3**	0.3371	0.4077	0.5657	0.5251	0.9003	0.7626
**Ki-67 day 15**	0.9709	0.4568	0.4177	0.9248	0.3957	0.9143
**Vinculin day 3**	0.1088	0.2598	0.7036	*0.0395*	0.1668	0.0609
**Vinculin day 15**	0.5392	0.2743	0.7225	0.1091	0.1531	0.1889

Numbers in *italics* indicate statistical significant difference. C1, C2, C3 and C4 indicate conditions 1, 2, 3 and 4.

### Glucose uptake showed high metabolic activity in C3 and C4 [n = 48, twenty four pairs]

It was observed that the average amount of glucose (µg/mL) that was taken up by day 3 in C1, C2, C3 and C4 was 0.34 (±0.33), 1.13 (±0.53), 2.13 (±0.36) and 2.0 (±0.27) respectively. This was statistically significant (p < 0.05) between C1–C4 and C1–C3 at day 3 (Fig. 2D). No other conditions showed any statistical significance at day 3. However, glucose uptake was statistically significant between all the groups (p < 0.05) except C1–C2 and C3–C4 at day 15 (Table 1) where C1, C2, C3 and C4 utilized 0.94 (±0.59), 1.33 (±0.58), 2.85 (±0.78) and 2.92 (±1.01) µg/mL of glucose (Fig. 2D). This means that the cells were functional throughout as the metabolism was active due to the consumption of glucose and production of lactic acid in all the groups. It was noticed that higher amount of glucose was uptaken in C3 and C4 by day 3 as compared to C1 and C2. This is because of the presence of HA and ROCK inhibitor. Faster cell adherence and start of proliferative phase was observed in C3 and C4. Both the groups have similar media composition, hence only the similar groups did not show any significance, which also highlights that apart from force adhesion using HA, ROCK is also important for higher cell adhesion and better proliferation.

### Hoechst, Calcein AM and Ehidium homodimer (HEC) staining showed higher number of cells at day 3 and confluency pattern at day 15 without any dead or apoptotic cells in C3 and C4 [n = 12, Six pairs]

**Figure 3:**
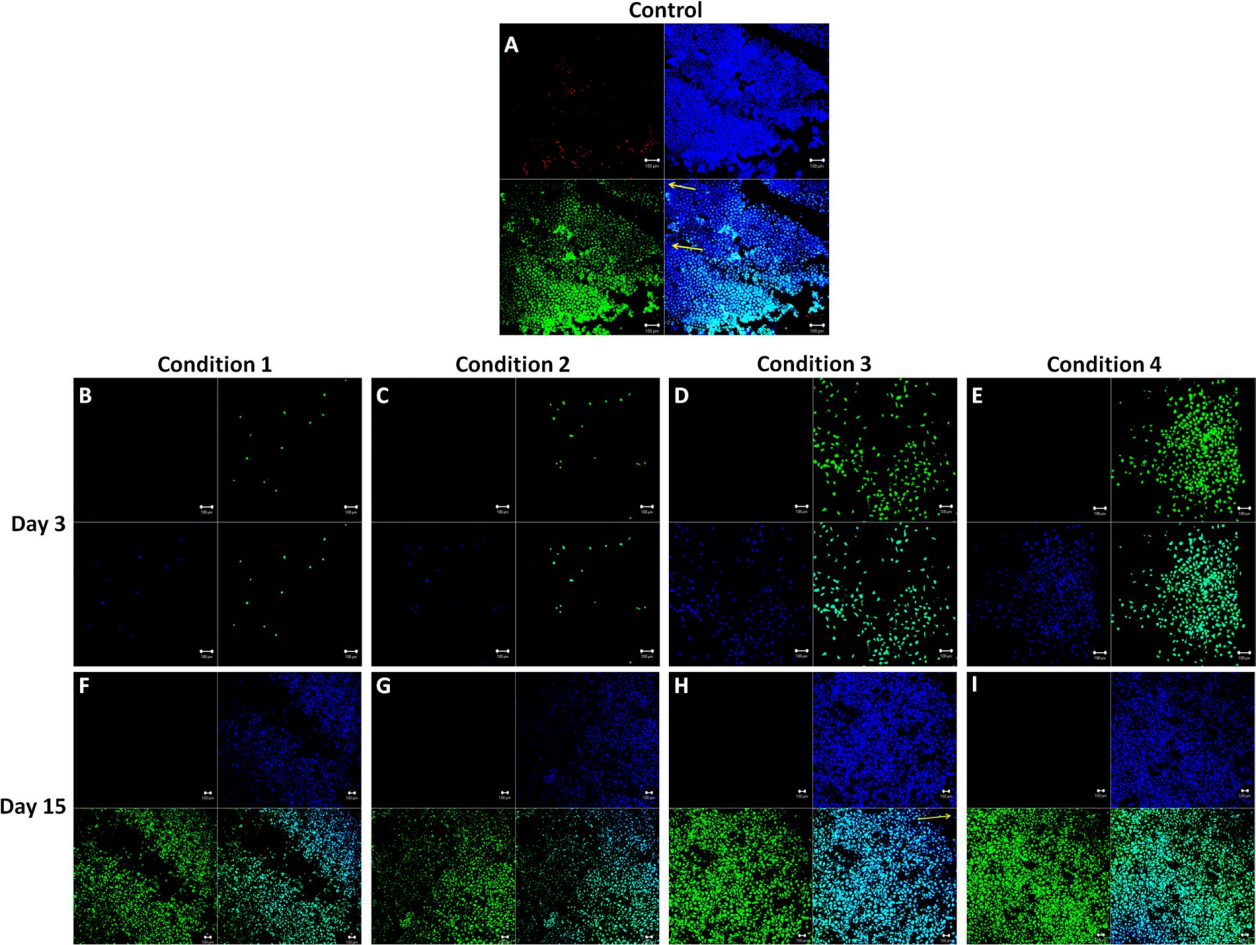
HEC staining to determine live/dead/apoptotic cells. (**A**) Control cornea to show the presence of dead (red), live (green), apoptotic (blue without green marked with yellow arrow) and nucleus (blue). (**B**–**E**) At day 3, higher number of cells was observed in C3 and C4 compared to C1 and C2 without any dead or apoptotic cells. F-I) at day 15 confluence of viable cells was observed in C3 and C4 compared to C1 and C2. Scale bar = 100 µm.

HEC was observed in the control group to show the presence of all the dyes for comparison (Fig. 3A). Yellow arrow marks the apoptotic cells, attached dying cells (without metabolic activity) to the extracellular matrix. At day 3, C3 and C4 showed more number of cells as compared to C1 and C2. Only viable cells were observed at day 3 (Fig. 3B–E). However, at day 15, confluency was observed in C3 and C4 compared to C1 and C2 with no dead cells and few apoptotic cells marked in yellow (Fig. 3H), which were not significant (Fig. 3F–I). This was a qualitative analysis and therefore statistics were not performed.

### Expression of Zonula Occludens-1 (ZO-1) and measurement of hexagonality and polymorphism showed significant data at day 15 [n = 12, Six pairs]

**Figure 4:**
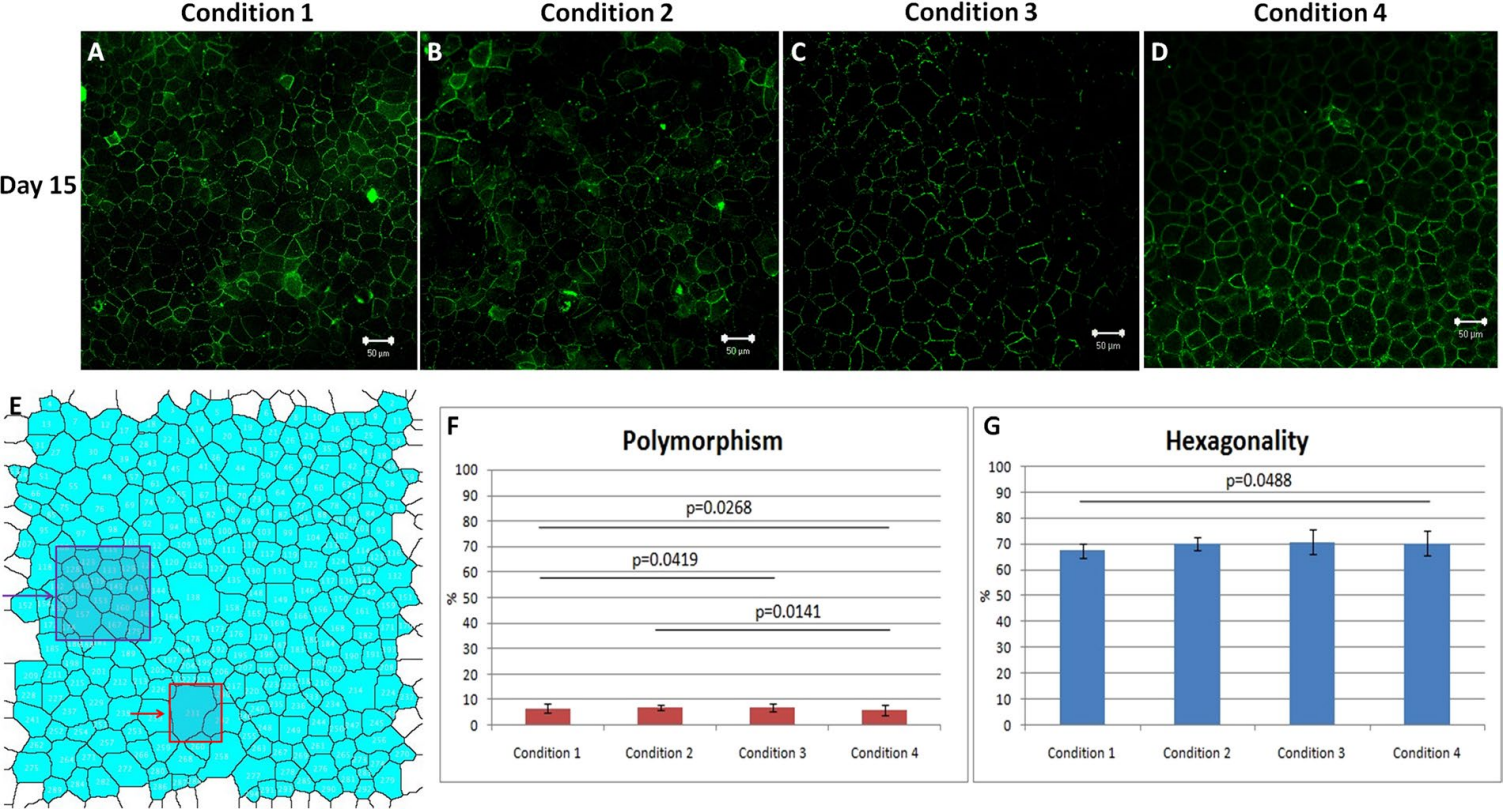
ZO-1 staining for intracellular tight junctions and to determine the polymorphism and hexagonality of the cells in different conditions. (**A**–**D**) The cultured HCEnCs showed expression of ZO-1 in all conditions. (**E**) The image was locked in imageJ and was processed to overlay masks for determination of hexagonal cells and number of cells that showed high polymorphism (polymegathism and pleomorphism). Purple block represent number of hexagonal cells and red block indicate highly polymorphic cell in the determined area. (**F**) Polymorphism was significantly lower in C4 and C3 compared to C1 and C2 with C1–C4 (p = 0.0268), C1–C3 (0.0419) and C2–C4 (p = 0.0141) showing less polymorphic cells in C3 and C4 compared to other conditions whereas (**G**) Hexagonality was significantly higher between C1–C4 (p = 0.0488) at day 15. Scale bar = 50 µm.

Zonula Occludens-1 (ZO-1) is a tight junction protein which is present in the intercellular borders between the cells. Apart from showing the functions of tight junction protein, it can also be indirectly use to judge the hexagonality of the cells. ZO-1 has also been widely used in the research of HCEnCs. At the end of the culture at day 15, ZO-1 (Fig. 4A–D) was expressed in all the conditions. Average value of hexagonality (%) (purple box in Fig. 4E) in C1, C2, C3 and C4 was 68.79 (±4.47), 70.58 (±2.18), 72.77 (±3.49) and 77.27 (±5.18) whereas the polymorphism (%) (indicated as red box in Fig. 4E) in the respective combinations was found to be 8.16 (±2.08), 6.86 (±1.05), 5.97 (±0.66) and 4.35 (±1.01). Higher polymorphism was observed between C1–C3 (p = 0.0419), C1–C4 (0.0268) and C2–C4 (0.0141) (Fig. 4F). Hexagonality showed significant difference between C1–C4 (p = 0.0488) (Fig. 4G). However, the cells from condition 1 and 2 showed slightly less hexagonal cells, which were checked using ImageJ (not significant, p > 0.05, Table 1, Fig. 4G) for other conditions. There was no significant difference between other conditions at the end stage (day 15) but as the cells were not fully confluent in C1 and C2, the cells showed higher polymorphism.

### Statistically insignificant expression of Ki-67 was observed in all the conditions [n = 12, Six pairs]

**Figure 5:**
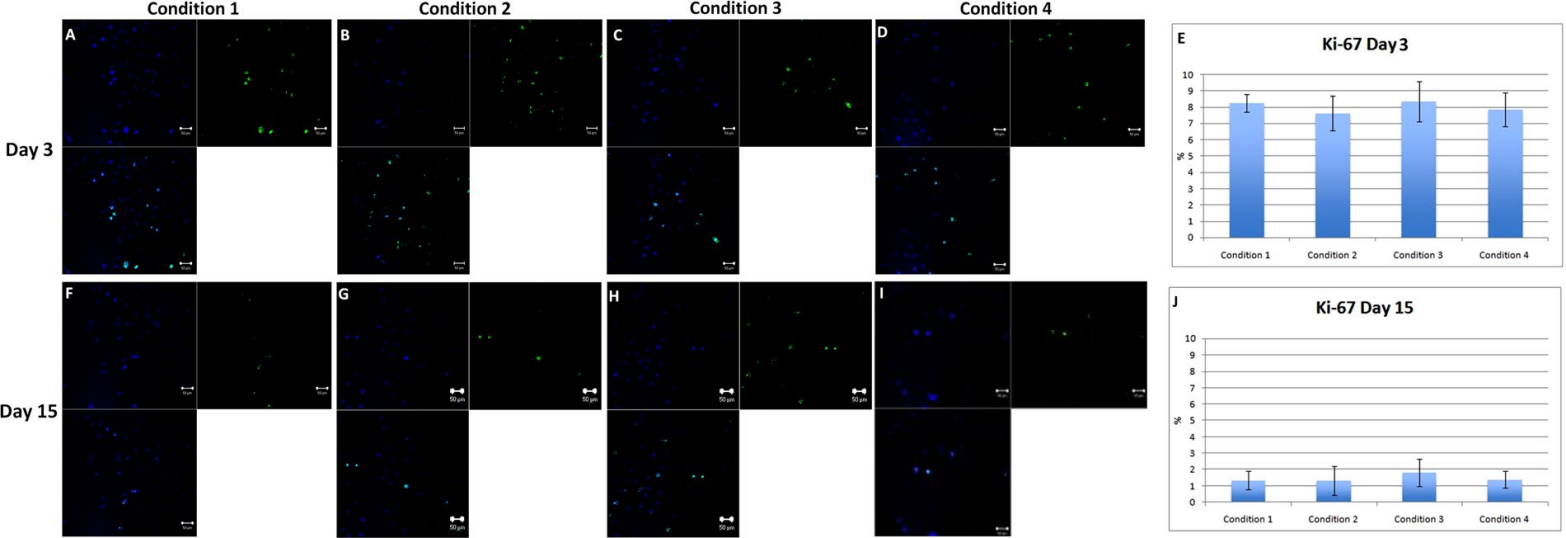
Expression of Ki-67, proliferative marker. (**A**–**D**) Ki-67 was almost equally expressed in all four conditions, which highlights that the plating density of proliferative cells was almost equal in all the conditions at day 3. (**F**–**I**) At day 15, number of Ki-67 cells decreased in all four conditions without any statistical significance. (**E**) Graphical representation of the number of cells that expressed Ki-67 at day 3 and (**J**) at day 15. Scale bar = 50 µm.

Ki-67 is a proliferative marker which is used to check the amount of proliferative cells present in the culture. Ki-67 was used at the initial stage to control the presence of nearly equal amount of proliferative cells in all the conditions and to rule out the possibility of a biased study with presence of higher number of cells in either condition. We performed this study on n = 3 separate tissues to optimize our plating density. It was observed that all the conditions had similar amount of proliferative cells at the beginning (day 3) (Fig. 5A–D) which decreased in number by day 15 (Fig. 5F–I), but did not show any significance (p > 0.05) (Table 1) in either condition. Average Ki-67 expression (%) found on day 3 in C1, C2, C3 and C4 was 8.25 (±0.53), 7.63 (±1.04), 8.34 (±1.22) and 7.86 (±1.02) (Fig. 5E) whereas on day 15, Ki-67 decreased to an average of 1.33 (±0.58), 1.32 (±0.90), 1.80 (±0.82) and 1.37 (±0.51) respectively (Fig. 5J).

### Vinculin showed higher focal adhesion points in C4 at day 3 [n = 12, Six pairs]

**Figure 6:**
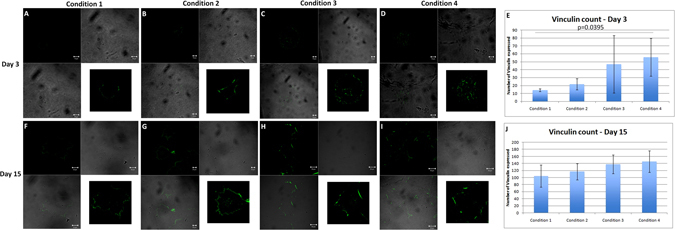
Expression of Vinculin as a focal adhesion marker. (**A**–**D**) Number of focal adhesions found in C4 was statistically higher compared to C1 (p = 0.0395). C3 also showed high number of focal adhesion points but was not significant at day 3. Scale bar = 10 µm. This highlights that mechanical and molecular forces may induce higher focal adhesions and integrins for better cell attachment. (**F**–**I**) At day 15, Vinculin was expressed in all the conditions without any significant difference. (**E**) Graphical representation of number of focal adhesions that expressed per cell on day 3 and (**J**) on day 15. Scale bar for C1 and C2 = 20 µm; C3 and C4 = 50 µm. Image inserts show a higher magnification of the analyzed cell with the expression of Vinculin.

Vinculin is a membrane-cytoskeletal protein in focal adhesion plaques that is involved in linkage of integrin adhesion molecules to the actin cytoskeleton. It is also associated with cell-cell and cell-matrix junctions, where it is thought to function as one of several interacting proteins involved in anchoring F-actin to the membrane. C1, C2, C3 and C4 showed 14.0 (±1.83), 21.75 (±6.95), 47.0 (±36.34) and 55.75 (±23.96) focal adhesion spots on day 3. C3 did not show any statistical significance compared to any groups (Fig. 6A–D). Vinculin was expressed in all the conditions at day 3 however; it was significantly expressed more in C4 compared to C1 (p = 0.0359) (Fig. 6E) (Table 1). No significant difference was found between any groups on day 15 (Fig. 6F–I) (Table 1) with 104.25 (±31.55), 117 (±22.85), 138 (±26.32) and 145.5 (±30.47) focal adhesion spots on day 15. The results highlight that higher focal adhesions may be induced with forced adhesion especially when there is an involvement of mechanical pressure because of HA and molecular force due to ROCK inhibitor (Fig. 6E,J).

## Discussion

Corneal endothelial dysfunction is a major indication for corneal transplantation^13^. However, the isolation and propagation of primary HCEnCs have been described by various research groups using different approaches^14–18^ to find alternative solutions reducing the global shortage of donor corneas. Cultivated HCEnCs derived from older donors have lower proliferative capability, a senescent cell phenotype, and cell morphology, which suggest less functional ability than those derived from younger donors^19^. If the HCEnCs can be cultured from old donor corneas, then the availability of donor corneas for isolating endothelial cells will increase and it will overcome the overall shortage of donor corneas available for transplantation^20, 21^.

Corneal endothelial dysfunction has been treated by full-thickness corneal transplantation (PK) since more than half a century, but more selective corneal endothelial replacement such as Descemet Stripping Automated Endothelial Keratoplasty (DSAEK) and Descemet Membrane Endothelial Keratoplasty (DMEK) were developed in the last decade^22, 23^. The clinically successful outcomes of DSAEK and DMEK imply that reconstruction of the corneal endothelium is a definitive treatment that can replace full thickness keratoplasty, further corneal endothelium reconstruction by cell-based therapy is a clinically relevant approach^24^. Currently, although HCEnCs are isolated from donor corneas and cultured for research purposes, several limitations such as limited proliferative ability, vulnerable transformation with loss of functions, and senescence that prohibits efficient *in vitro* expansion for clinical use have not been answered. No protocol specifically designed for clinical application has been established so far.

It is already known that inclusion of ROCK inhibitor Y-27632 elevates the adhesion property, enhances cell proliferation and suppresses apoptosis of HCEnCs, thus allowing the successful transplantation of HCEnCs to reconstruct functional corneal endothelium^25^. Another report has emphasized the effect of ROCK inhibitor in enhancing adhesion and wound healing of HCEnCs^7^. It has already been noted that Rho-ROCK signalling carries out a variety of cellular processes such as cell adhesion, morphogenesis, migration, and cell-cycle progression through mediating cytoskeletal dynamics. Rho regulates a variety of cytoskeletal dynamics that underlie cell morphology and adhesion through the activation of ROCK^26, 27^. The low efficiency of engraftment and loss of phenotype after transplantation due to the absence of cell/cell and cell/ECM interaction *in vivo* impairs organ reconstruction in various tissues^28–30^. Researchers have therefore been exploring the use of various techniques, such as artificial scaffolds, biologically active molecules, and ECM coatings, to improve cell retention and survival^31–33^.

Hyaluronan contributes significantly to cell proliferation and migration and may also be involved in the progression of some malignant tumors. Cell migration is promoted by HA using its physiochemical properties along with its direct interaction with cells. HA provides an open hydrated matrix that facilitates cell migration^34^. Directed migration and control of the cell locomotory mechanisms are mediated via specific cell interaction between HA and cell surface HA receptors that are CD44, RHAMM, and ICAM-1. It has been noted that RHAMM is more related to cell migration. It forms links with several protein kinases associated with cell locomotion^35–37^. By providing the dynamic force to the cell, HA synthesis has also been shown to associate with cell migration^38^. A schematic diagram showing the adhesion of cells with hyaluronic acid (Fig. 1A) along with the images as seen in the microscope of the unattached and attached cells (Fig. 1B) are represented. Chondroitin sulphate is a sulphated glycosaminoglycan composed of a chain of alternating sugars. It is commonly used in corneal preservation medium and has shown positive effects in preservation of corneal endothelial cells in hypothermic conditions. The presence of chondroitin sulphate along with hyaluronic acid makes the mixture denser and additionally branched because of its structure. This makes a strong net further attaching to maximum number of cells and allowing them to precipitate and stick to the base of the plastic due to forced adherence. Higher adherence is associated with better proliferation and therefore hyaluronic acid seems to be a better solution to increase cell attachment.

Although the forced adhesion technique is relatively robust, the HCEnC cultures can be affected substantially by the procurement process as well as donor variations. The characteristics that we have used for this study are extreme with old donor age, long preservation time and endothelial cell count that are less than the normal density required for transplantation. If these challenging cells can be cultured in the appropriate conditions then the cells from young donors with high cell density and proliferative capability will definitely show increased proliferation rate (also internally observed in one donor – 32 year old). Donor characteristics such as health of the donor before death, the cause of death, as well as the duration from death to enucleation and preservation and the time from preservation to the establishment of culture^39–42^ has also shown to have a potential impact on the cell culture. Beyond these potential hindrances, we report herein that the combination of HA and ROCK (C4)^43^ approach in expansion of HCEnCs can be further enhanced, in terms of high adhesion and proliferation capability. The shorter time it took for HCEnCs to reach confluence was most likely due to the increased proliferation rates as observed in C4 due to high adhesion rate. It has been reported that the use of Y-27632 to increase the proliferation of HCEnCs may only be appropriate for cultures of HCEnCs established using younger donors, as the addition of Y-27632 were not found to be advantageous for cultures established using older donors^12^. Similar observation has been also reported that Y-27632 has no effect on the proliferative capacities on HCEnCs, which utilized corneas from donor ages 73 ± 3 years^7^. However, forced adhesion of the HCEnCs from older donor corneas may show a potential impact in cell culture of these low proliferative cells.

In this study, we investigated the differences in cellular phenotype in response to culture in different types of adherence conditions. We found that the proliferative capability of HCEnCs differed depending on the conditions used. Four conditions were selected to check the adhesive properties and proliferative capabilities especially for older donors. The isolation of HCEnCs is very much important as it determines the preliminary success of the culture. If the isolation is not performed correctly, it may lead to contamination of stromal fibroblasts or trabecular meshwork cells. We did observe such contaminations in our very first cultures (excluded from this study). Gentle peeling of Descemet membrane and endothelium piece-by-piece seems to be the best solution so far. We have previously tried using a Descemet Membrane Endothelial Keratoplasty (DMEK) donor punch (9.5 mm). This has a great advantage in terms of excising two exclusive parts of the endothelium (periphery and centre) with cell loss that only occurs at the punch site. However although punching method can be used, there is a possibility that increase in punching pressure may pierce the stroma which may then activate keratocytes leading to unwanted contamination of the fibroblasts. Peeling a larger diameter DMEK graft (9.0 mm) and incubating it with Collagenase type 1 also takes longer time for the cells to digest due to the less available area for the enzyme to cleave between the cells. It was also noted that the DMEK tissue of the younger donors are firmly adhered to the stroma as compared to the older donors which can be peeled easily if the donors have no other contraindications like diabetes. Therefore manual peeling of the cells piece by piece was determined as a suitable protocol for this study.

It has been widely reported that HCEnCs isolated from older donors are less proliferative than those established from younger donors, and this has been associated to significant increase of cyclin kinase inhibitors, hence resulting in an age-dependent increase in negative regulation of cell cycle^44^. However, using C4 i.e. HA combined with ROCK, it is possible to culture the older donor corneas too with a high proliferative capability. The presented data highlighted that the cells can reach confluence by day 10 when provided with conditions like C4. We used 15 pairs of older donor corneas and the confluency pattern seen was significant between C1 and C4 suggesting that C4 was superior in terms of cellular proliferation. However, C3, the condition with only ROCK did not show any significance in confluency with C4 or C1 but showed lesser proliferative capability than C4 up to a week. Moreover, all the tissues that were collected for this study had an endothelial cell density <2200 cells/mm^2^. The plating density of younger donor corneas is around 550,000 cells/9 cm^2^, which is less than the plating density we have used which is around 93,000 cells/0.7 cm^2^. Increasing the plating density for <1 cm^2^ area (optic zone) could also be sufficient for culturing older donor corneas. Moreover, the plating density recorded was 93000 cells/well (which is around 2000 cells/mm^2^). However, at the end of confluency, we recorded approximately 2400 cells/mm^2^, which if recalculated comes to around 120,000 cells/well. The amount of cells now falls under a transplantable category for PK (from 2000 cells/mm^2^ to 2400 cells/mm^2^) which was initially not suitable for transplantation.

We have noticed that relating culture conditions, proliferation and confluency patterns to donor characteristics was very difficult as some donors show good proliferation rate and others don’t. However, most of the donors were cultured with success using C3 and C4. Morphological analysis showed hexagonal cells when cultured in different conditions and very less amount of cells <10% showed high polymorphism. We were able to culture 4 wells (0.7 cm^2^ each), which are similar to an optic zone by pooling low-density old age donor corneas and get them to confluence with good morphology and high rate of hexagonality. As it is difficult to obtain younger donor corneas with high-density endothelial cells, the technique (C4) could be of valuable importance in terms of donor availability and culture capacity. Moreover, if the target is to culture primary endothelium into multiple grafts, then high plating density in combination with C4 could be a valuable solution especially when the tissues are isolated from older donors. With this approach, there is a practical possibility of obtaining 4 grafts from 2 corneas (1 donor unsuitable for transplantation) as shown in the paragraph above.

The amount of utilized glucose can be determined during the culture by checking the metabolic activity of the cells. When the cells metabolize, they produce lactic acid from glucose and therefore the amount of glucose uptaken in the media by the cells can be evaluated. In this study we show that the amount of glucose that was utilized in the first 3 days was significantly higher in C4. This was due to a higher number of proliferating cells in the first three days. However, when the cells were confluent at day 15, all the conditions showed insignificant amount of glucose uptake, which confirmed that majority of the cells were metabolically active during the culture stage and at the confluent stage. If the cells were stressed due to the acidity levels of HA, they would have also shown high glucose uptake in C2, which was not the case. It only showed in C3 and C4, which further highlights that HA are safe to be used for cell culture.

HEC staining also showed that the cell proliferation in C3 and C4 was higher compared to C1 and C2 at day 3 and day 15 when it was confluent. A minimum amount of apoptotic cells were observed with no dead cells in the culture. The cell viability was higher in all the conditions. ZO-1 immunostaining was used to check the hexagonality and the presence of tight junctional proteins. It was observed in separate conditions (data unpublished) that ZO-1 did not express much when the cells were packed because even though the cells look confluent, the time required for the development of tight junctional protein is higher. Therefore, the cells should be allowed to culture for an extra day after confluency just to ensure that they have properly developed intra-cellular tight junctions. Cell shape is related to various cell functions, such as the communication with other cells, regulation of cell movement. Polymorphism was not high in the cells cultured in either condition at the day of confluency as all the conditions utilize ROCK inhibitor, they all proliferate up to the confluence stage. Condition 4 showed lower polymorphism as compared to the other conditions. Force adherence helps to maintain the cell shape and size. To ensure the plating density of the proliferative cells is almost the same in all the conditions, we optimized our plating conditions in different set of corneas before the experiment. It was observed that the proliferative cells found in different conditions were almost the same in all four conditions at day 3. Proliferative cells showed a decline in number at day 15 in all the conditions which is already previously known that the proliferative capabilities of the cells decreases with time.

A previous study shows that actin cytoskeleton plays a critical role in regulating the adhesive property through interaction between the actin cytoskeleton and integrin^45–47^. Another study reported that inhibition of ROCK signalling by a selective ROCK inhibitor or by the siRNA enhances adhesive property of corneal endothelial cells and is consistent with the findings of those previous studies. It is also found that vinculin, which is involved in the linkage of the integrin adhesion complex to the actin cytoskeleton^48, 49^ is upregulated in ROCK-inhibitor treated HCEnCs. However, it is noted that further investigation is required to elucidate whether the ROCK inhibitor promotes the focal adhesions through inhibiting actin polymerization and induces the upregulation of cell adhesion properties on the extracellular matrix (ECM)^5^. Vinculin expression and HEC staining revealed the same thing, that C3 and C4 show higher number of cells at day 3 and day 15. C1 and C2 showed lower number of cells when compared to C3 and C4. Vinculin expression was significantly higher in C4 at day 3. Therefore, it is assumed that force adherence in combination with ROCK may be responsible to induce more focal adhesions and hence integrins and expression of Vinculin.

Due to the availability of young donors for research, we were only successful in culturing one young donor. We observed confluency on day 7 with high hexagonality (approximately 95%) when young donors (age 32) with high plating density were cultured using forced adhesion with HA and ROCK. Old donors may not have a high proliferative capability but if they are forced to attach and proliferate, although it may take longer to confluence, they do proliferate and manage to obtain the required cell size and shape.

A surgical hypothesis for using HA is that it is currently used in the anterior chamber surgical procedures. The injection technique for introducing cultured HCEnCs with face down position is already been studied by Kinoshita and colleagues (data unpublished). The patients have to rest their face down for 2–3 hours which may be longer for some patients therefore, introducing HA after the delivery of HCEnCs and face down position may help the cells to adhere to the stroma faster compared to the regular adherence speed. This may have a potential clinical impact in the future.

In conclusion, the findings of this present study, which are supported by the previous data, indicate that ROCK inhibitor Y-27632 in combination with HA may enable the establishment of a cultivated-HCEnC–based therapy. This novel strategy of using a force attachment of the HCEnCs combined with a ROCK inhibitor on older donor corneas with less proliferative and isolated cells may ultimately provide clinicians with a new therapeutic modality in regenerative medicine and reduce the global shortage of the donor corneas for the treatment of endothelial disorders.

## Material and Methods

### Ethical Statement

The corneas [n = 48, twenty four pairs] were collected from the Veneto Eye Bank Foundation (FBOV) with written consent from the donor’s next of kin to be used for research. The methods were carried out in accordance with declaration of Helsinki. The tissues were used under the laws of CNT (Centro Nazionale di Trapianti). The corneas were suitable for research and unsuitable for transplantation due to low endothelial cell count (<2200 cells/mm^2^). No other complications or indications were noted in the donor corneas. All the tissues were preserved in tissue culture medium at 31 °C prior to use.

### Endothelial cell count and donor characteristics

Endothelial cell density (ECD) and mortality before isolation was checked using trypan blue (TB) stain (0.25%) (Thermo Fisher Scientific (Rochester, NY, USA), which is routinely used in eye banks. Approximately 100 µL of TB was topically applied to stain the endothelial cells for 20 seconds followed by washing it with phosphate buffered saline (PBS). Trypan blue positive cells (TBPCs) were recorded before isolation using an in-built eyepiece reticule for inverted microscope (Axiovert, Zeiss, Germany). The eye-piece reticule was also used to check the number of cells before isolation and after confluence. The donor characteristics of 48 corneas (twenty four pairs) were obtained from the FBOV database to determine the age, gender, post-mortem time, cause of death, preservation time, ECD and mortality.

### Sodium Hyaluronate

Sodium hyaluronate is a naturally occurring, high molecular mass, polysaccharide found in the extracellular matrix of the connective tissues. Sodium hyaluronate, and other viscoelastic substances including methylcellulose, chondroitin sulphate, polyacrylamide, and collagen, have been used in intraocular surgery since 1970s^50^. In this study, we used Viscoat (Alcon, Texas, USA) −0.8 mL comprising of 3% sodium hyaluronate and 4% chondroitin sulphate with Resting State Viscosity of 40,000 ± 20,000 cps that is currently being used in corneal and cataract surgery. The viscosity of HA allows the cells to settle down faster and force the cells to adhere to the coated base quicker.

### Formulations

The procedure of isolation and culture was slightly modified from the previously published article by Peh *et al*.^12, 39^. Two different types of media were used as described earlier^39^, which is maintenance media and proliferative media. In brief, the maintenance medium (M5) contained human endothelium SFM, 5% FBS along with antibiotics. The proliferative medium (M4) was a mixture of HamF12, M199, 5% FBS, 1% ascorbic acid, 0.5% Insulin Transferrin Selenium (ITS), Rec human FGF basic (25 µg/mL), 10 µM ROCK inhibitor (Y-27632) and antibiotics.

### Peel and digest method

The method is similar to previously published article^39^ with some modifications which is described in brief. The corneal tissues [n = 48] were washed in sterile phosphate buffered saline (PBS) and the Descemet membrane-endothelial tissues were peeled gently (similar to the stripping technique for Descemet membrane endothelial keratoplasty – DMEK method) in various pieces to ensure faster isolation of the cells when incubated in the enzyme. The excised pieces were incubated with 2 mg/mL Collagenase Type 1 (Thermo Fisher Scientific, Rochester, NY, USA) solution for 2–3 hours at 31 °C and 5% CO_2_. While peeling, the forceps was cleaned using PBS after every single encounter with Collagenase Type 1 solution. Once the cells were isolated from the Descemet membrane, they were collected with Collagenase Type 1 and centrifuged for 5 minutes at 1000 rpm in a 15 mL falcon tube. The supernatant was removed and the cells were re-suspended with TrypLE Express (1X), phenol red [Life Technologies, Monza, Italy] to cut the clumps into single cells for 10 minutes at 37 °C [if the clumps were not converted to single cells then TrypLE Express treatment was repeated for 5 minutes]. The supernatant was removed and the cells were re-suspended with 400 µL of M5 after counting them with TB in a haemocytometer slide. The plating density was recorded for all the cultures.

### Plating and force attaching the cells on coated slides

Labt-Tek II chamber slides (8 chambers, 25 × 75 mm, 0.7 cm^2^ culture area) from Thermo Fisher Scientific (Rochester, NY, USA) was used for plating the cells. All the chambers were coated with 50 µL FNC coating mix [Cell attachment Reagent (FNC Coating mix) BRFF AF-10, US Biological Life Sciences, Salem, Massachusetts, USA] for at least 30–45 minutes at 37 °C, 5% CO_2_. The residual coating was removed before the plating. 100 µL of the cell suspension was mixed well and added in each chamber to ensure equal distribution of cells including proliferative cells in all the conditions. Four chambers were used for each donor that was pooled together. The chamber slides were used to check four conditions as shown in Table 2. The cells were monitored every alternate day till confluence.

**Table 2:** Description of plating conditions.

Condition 1	Condition 2	Condition 3	Condition 4
100 µL of cell suspension + 200 µL of M5 up to day 3.	100 µL of cell suspension + 200 µL of M5 up to day 3.	100 µL of cell suspension + 200 µL of M4 throughout the culture period. The media was refreshed every alternate day.	100 µL of cell suspension + 200 µL of M4 throughout the culture period. The media was refreshed every alternate day.
The media was changed after day 3 to M4 and was re-freshed every alternate day	Cells were force adhered using gentle addition of Viscoelastic (HA) (approximately 50–100 µL) on top of the cell suspension.		Cells force adhered using gentle addition of Vicoelastic (HA) (approximately 50–100 µL) on top of the cell suspension.
	The media was changed after day 3 to M4 and was re-freshed every alternate day		

Condition 1, 2, 3 and 4 shows different plating characteristics with and without HA and ROCK. In condition 1, no HA or ROCK was used to check the behaviour of the cells without the influence of these extra parameters. In Condition 2, only HA was added to check the adherence of the cells without ROCK. Condition 3 only utilized ROCK inhibitor from the day of plating to check the effect of ROCK inhibitor directly on the cells. Condition 4 was a mixture of HA and ROCK to understand the influence of both the parameters together.

### Morphological analysis of the cultured HCEnCs [n = 30, fifteen pairs]

The cells were visualized every alternate day until confluence using an inverted microscope (Axiovert, Zeiss, Germany) at 50X and 100X magnifications. The percentage confluency was monitored and recorded every alternate day manually using an eyepiece reticule.

### Glucose uptake of the cultured HCEnCs for functionality checks and metabolic analysis [n = 48, twenty four pairs]

Glucose uptake was determined from the preservation media of all the samples at day 3 and day 15. The amount of glucose used and lactic acid produced at day 3 (with adapting and proliferative cells) and day 15 (confluent cells) were recorded. The assay helps to check the metabolic activity of the HCEnCs when preserved *in vitro*. Quantitative analysis was performed using D-Glucose HK kit (Megazyme International Ireland Ltd, Bray Business Park, Bray, Co. Wicklow, Ireland).

For Hoechst, Calcein AM and Ehidium homodimer (HEC) and, Immunostaining of Ki-67 and Vinculin, the study was divided into two groups, a) n = 6 (three pairs), to evaluate the early behaviour of the cells at day 3 and b) n = 6 (three pairs), to evaluate the nature of confluent cells at the end of the study. ZO-1 was only evaluated at the end of the culture period (day 15).

### Hoechst, Calcein AM and Ehidium homodimer (HEC) staining to determine live, dead and dying cells [n = 12, Six pairs]

Live cells are distinguished by the presence of intracellular esterase activity, determined by the enzymatic conversion of the nonfluorescent cell-permeant Calcein AM to the intensely fluorescent Calcein. Calcein, is retained within live cells, producing an intense green fluorescence in live cells. Ethidium Homodimer-1 (EthD-1) enters cells with damaged membranes and enhances fluorescence upon binding nucleic acids, thereby producing a bright red fluorescence in dead cells. EthD-1 is excluded by the intact plasma membrane of live cells. Hoechst 33342 is a nuclear dye which stains the nucleus of the cell. Triple endothelial labelling with Hoechst 33342 (H), Ehidium Homodimer (E), and Calcein-AM (C) showed expression of ‘E’ in red representing the dead cells, blue represents the nuclei ‘H’ and green marked the living cells ‘C’.

The cells were washed with PBS prior to the assay to remove or dilute serum esterase activity generally present in serum-supplemented growth media such as M4 or M5. Control sample was prepared using a human donor cornea. Descemet membrane was peeled out using stripping method and the excised lenticule was washed before the assay. Some cells were damaged by purpose through gently scrapping and touching the endothelial cells using sharp forceps. 5 µL of Hoescht 33342 (H) (Thermo Fisher Scientific, Rochester, NY, USA), 4 µL of Ethidium Homodimer EthD-1 (E), 2 µL Calcein AM (C) (Live/Dead viability/cytotoxicity kit, Thermo Fisher Scientific, Rochester, NY, USA) was mixed in 1 mL of PBS and mixed well. 100 µL of the final solution was directly added on the cultured cells and incubated at room temperature in dark for 45 minutes. For the control tissue, the stripped lenticule was placed on the slide, cut at four peripheral sides for a flat mount and covered with coverslips without the mounting medium. HEC was viewed at 488, 495, 515 and 635 nm at 50X, 100X and 200X magnification.

### Immunostaining for tight junctions using Zonula Occludens-1 (ZO-1) [n = 12, Six pairs], proliferation marker Ki-67 [n = 12, Six pairs] and focal adhesion marker Vinculin [n = 12, Six pairs]

The cells were washed with PBS and fixed in 4% paraformaldehyde (PFA) at room temperature (RT) for 30 minutes. The cells were permeabilized with 0.5% Triton X-100 in PBS for 30 minutes. After blocking with 2% goat serum for 2 hours at RT, the tissues were incubated overnight at 4 °C with primary antibodies [anti-ZO-1, 1:200 (ZO1-1A12, Thermo Fisher Scientific, Rochester, NY, USA); anti-Ki-67, 1:200 (MIB-1, Milan, Italy); anti-vinculin, 1:200 (Abcam, Cambridge, Massachusetts, USA). The samples were incubated with goat anti-mouse fluorescein isothiocyanate (FITC)-conjugated secondary antibody in 20% goat serum for 2 hours at RT. After each step, the cells were washed 3 times with 1X PBS. After removing the wall of the Lab-Tek slide, the cells were covered with mounting medium and cover slips. Cells were examined with an LSM 510-meta laser scanning microscope (Zeiss, Milan, Italy). Cells were examined under ultraviolet light or by excitation at wavelengths of either 488 nm or 594 nm where subsequent detection of the fluorescence was obtained.

### Measurement and statistical analysis

ImageJ (FIJI) software was used to measure the number of endothelial cells and check the hexagonality and polymorphism of the cells showing expression with ZO-1. It was also used to check the percentage of Ki-67 positive cells in the given area and to check the number of focal adhesions (Vinculin positive cells). For Vinculin, 10 cells per combination were analyzed. Each cell was separated and the backgrounds were subtracted. The area was measured and the threshold was applied further to convert the entire cell into binary masks. The particles were analyzed using outline option and watershed was applied if necessary. For ZO-1, the area was selected and using pre-defined commands in Macros for converting the image to overlay masks, the total number of cells was automatically counted whereas the hexagonal cells and polymorphic cells were counted based on the cell structure in the particular area. The Macros was designed particularly for this study to obtain results by simply inserting the algorithm in the ImageJ analysis.

ANNOVA and non-parametric Wilcoxon test for paired data using SAS statistical software was employed to check the statistical significance between different conditions where p < 0.05 was deemed significant.
